# Synthesis and Characterization of Photoluminescent Ce(III) and Ce(IV) Substituted Hydroxyapatite Nanomaterials by Co-Precipitation Method: Cytotoxicity and Biocompatibility Evaluation

**DOI:** 10.3390/nano11081911

**Published:** 2021-07-25

**Authors:** Andrei Viorel Paduraru, Adina Magdalena Musuc, Ovidiu Cristian Oprea, Roxana Trusca, Florin Iordache, Bogdan Stefan Vasile, Ecaterina Andronescu

**Affiliations:** 1 Faculty of Applied Chemistry and Materials Science, Department of Science and Engineering of Oxide Materials and Nanomaterials, University “Politehnica” of Bucharest, 060042 Bucharest, Romania; viorel.paduraru@upb.ro (A.V.P.); amusuc@icf.ro (A.M.M.); ovidiu.opre@upb.ro (O.C.O.); truscaroxana@yahoo.com (R.T.); ecaterina.andronescu@upb.ro (E.A.); 2 National Centre for Micro and Nanomaterials, University “Politehnica” of Bucharest, 060042 Bucharest, Romania; 3 “Ilie Murgulescu” Institute of Physical Chemistry, Romanian Academy, 060021 Bucharest, Romania; 4 Faculty of Veterinary Medicine, Department of Biochemistry, University of Agronomic Science and Veterinary Medicine, 011464 Bucharest, Romania; floriniordache84@yahoo.com; 5 National Research Centre for Food Safety, University “Politehnica” of Bucharest, 060042 Bucharest, Romania

**Keywords:** hydroxyapatite, photoluminescence, cerium ions, biocompatibility evaluation

## Abstract

Improved compounds of Ce(III) and Ce(IV)-doped hydroxyapatite (Ca_10-x_Ce_x_(PO_4_)_6_(OH)_2_) with different concentrations such as x = 0.5, 1, 2.5, 5, and 10%, obtained by the simple co-precipitation method were synthesized. The cerium (3^+^) and cerium (4^+^)-doped hydroxyapatite were evaluated for biocompatibility and fluorescence properties. It was found that the cerium-HAp powders were non-toxic, even at higher level of concentration. The synthesized powders were further characterized by FTIR spectrometry, UV-Vis spectroscopy, XRD diffraction, SEM and TEM analysis. Therefore, the present study proves that the developed cerium (3^+^) and cerium (4^+^)-doped hydroxyapatite, respectively can be widely used as luminescent labeling materials, with improved biological properties.

## 1. Introduction

In the world of bioceramics, chemical compounds such as calcium apatites and other calcium orthophosphates are widely used in many fields of science, including geology, chemistry, biology, and medicine due to their abundance and presence in living organisms [1]. Bioceramics can be defined as a biocompatible ceramic material, manufactured in a porous, dense, amorphous or crystalline form, which can be used for the repair and reconstruction of different parts of tissue. The main group of bioceramics is derived from calcium phosphate (CaP), the others are zirconium (ZrO_2_), alumina (Al_2_O_3_), carbon, silicates, and phosphate ceramics [2].

Photoluminescence is an important and useful mechanism for *in situ* investigation of tissues, fluorescent elements being used in clinical trials for years. Recently, various studies used nanoparticles or different components for this purpose [3].

Hydroxapatite (HAp) is the main inorganic compound of human hard tissue [4]. The composition, crystal size, morphology, and stoichiometry of biological apatite are different from synthetic HAp. The Ca/P molar ratio for HAp is 1.67 [5,6]. An important application of HAp is in the treatment with chemotherapeutic drugs and antibiotics, mainly due to its porous surface and highly biodegradable properties [7]. Various studies suggest that calcium phosphate nanoparticles can be successfully used as a fluorescent probe after doping with lanthanide [8,9]. The main methods used to obtain hydroxyapatite are: Conventional method [10,11], wet chemical synthesis [12], hydrothermal conversion of calcium carbonate [13,14], bone calcination [15,16], solid phase reactions [17,18], co-precipitation reactions [19,20], sol-gel method [21,22,23], biomimetic synthesis [24,25], and pyrolysis [26,27].

Rare earth elements (REEs) are the common term used to refer to the elements from lanthanum to lutetium (atomic number 57 to 71). REEs have received increasing attention in the past decade since they can be used in various applications, such as catalysts, magnets, alloys, ceramics, electronics, etc. [28].

Cerium (Ce, atomic number: 58) is the second and most abundant element of the lanthanide series. While it shows the 3^+^ oxidation state characteristics of the series, it can be distinguished among the lanthanides by its unique ability to be oxidized to the 4^+^ state [29]. Recent studies have shown that cerium salts may stimulate metabolism. Researches have investigated the biological properties of cerium oxide and it has been proven that it can induce angiogenesis through its direct effect on the modulation of oxygen in intracellular environments. The antibacterial properties have also been studied, especially of strains on *E. coli*, *B. subtilis*, *Salmonella typhimurium,* and *Enterococcus faecalis*. The antibacterial properties of cerium ions make cerium a proper material to be used in biomaterials engineering. However, the literature contains reports of the synergistic effect of the activity of cerium ions in nanomaterials [30].

In living cells research, biological probes doped with lanthanide elements have proven that they are the most effective biological tools for staining and diagnostics [8]. In the field of nanotechnology, there is a growing trend in research with applications in healthcare. Currently, rare earth-doped nanoparticles are being studied for luminescent optical activity, being a nonisotopic substitute for organic fluorophores. The application of this class of materials may vary from *in vivo* detection of cellular function and luminescent labeling of biologically related molecules to the clarification of the structure and function of proteins and enzymes [31].

Only a few works in the literature are dedicated to HAp doped with cerium ions [32,33,34,35,36]. Although studies have been conducted by the researcher to the synthesis of the HAp substituted only with cerium(III), the problem is still insufficiently explored. Our study can be considered as a next step towards a more profound understanding of the substitution of calcium ions from HAp lattice with Ce^3+^ and Ce^4+^ ions. This work provides a new solution in further studies for the use of cerium substituted HAp for biomedical applications.

The main goal of this study is to demonstrate the application of the obtained cerium(III) and cerium(IV)-doped hydroxyapatite by the co-precipitation method as fluorescent probing for cellular imaging. The present research analysis is conducted through the variation of ion concentration ranging from 0 to 10%, the influence in structural and luminescence properties of the hydroxyapatite powders. Moreover, the differences and the influences of the two Ce^3+^ and Ce^4+^ ions on the crystalline structure, morphology, surface characteristics, and the photoluminescence properties were also investigated.

## 2. Materials and Methods

### 2.1. Chemical Materials

Calcium nitrate tetrahyrate (Ca(NO_3_)_2_·4H_2_O, 99.9%, Sigma-Aldrich, St. Louis, MO, USA), cerium-(III) nitrate hexahydrate (Ce(NO_3_)_3_·6H_2_O, 99.0%, Alfa Aesar, Haverhill, MA, USA), ammonium cerium (IV) nitrate ((NH_4_)_2_Ce(NO_3_)_6_, 98.0%, Alfa Aesar, Haverhill, MA, USA), ammonium phosphate dibasic [(NH_4_)_2_HPO4, 99.0%, Alfa Aesar, Haverhill, MA, USA), ammonium hydroxide (NH_4_OH, 25% solution, Alfa Aesar, Haverhill, MA, USA) were of analytical grade and used without further purification. The experiments were performed in deionized water.

### 2.2. Syhthesis of Pure HAp, Ce^3+^-Doped Hydroxyapatite and Ce^4+^-Doped Hydroxyapatite by the Co-Precipitation Method

Notation: HAp—the undoped hydroxyapatite, Ca_10−x_Ce(III)_x_(PO_4_)_6_(OH)_2_ and Ca_10−x_Ce(IV)_x_(PO_4_)_6_(OH)_2_ represent the hydroxyapatites doped with low ions concentration of Ce^3+^ and Ce^4+^, respectively, where x = 0.05, 0.1, 0.25, 0.5, and 1.

The HAp and cerium-doped hydroxyapatite nanoparticles with various contents of cerium ions were synthesized by the co-precipitation method. The method was previously described [37]. The pure HAp was prepared by adding dropwise of Ca(NO_3_)_2_·4H_2_O aqueous solution to an appropriate amount of ammonium phosphate dibasic aqueous solution to achieve the Ca/P atomic ratio of 1.67, under magnetic stirring for 2 h, adjusting and maintaining the pH of the resulting suspension at 10.5, by adding the NH_4_OH (25%) solution. The cerium-doped HAp powders (cerium (III) and cerium (IV)) were obtained by adding drop-wise a solution of ammonium phosphate dibasic to the solutions obtained by dissolving an appropriate amount of Ca(NO_3_)_2_·4H_2_O with different reagent grades of cerium (cerium-(III) nitrate hexahydrate or ammonium cerium (IV) nitrate) in deionized water, under vigorous stirring at room temperature. The Ce^3+^ and Ce^4+^ were taken in various concentrations such as 0.5, 1, 2.5, 5, and 10% and varied the Ca^2+^ content to maintain the stoichiometric ratio (Ca + Ce)/P of 1.67 (Ca_10−x_Ce(III)_x_(PO_4_)_6_(OH)_2_). The reaction was kept under continuous stirring for 2 h, adjusting the pH of the suspension at 10.5, by adding the NH_4_OH (25%) solution. The suspensions were kept for 24 h. After maturation, the precipitates were filtered off, washed with deionized water, at a pH value close to 7.0. The obtained powders were dried in an air oven at 80 °C for 12 h.

### 2.3. Sample Characterization

The morphological, structural, and optical properties of the obtained samples were investigated by means of Fourier Transform Infrared spectrometry (FT-IR), scanning electron microscopy (SEM), bright field transmission electron microscopy (TEM), X-ray diffraction (XRD), UV-Vis and photoluminescence (PL) spectroscopy. The FTIR spectra of all the samples were recorded using a Nicolet iS50R spectrophotometer (Thermo Fisher, Waltham, MA, USA), at room temperature, with ATR mode at 4 cm^−1^ resolution, in the measurement range 4000–400 cm^−1^. The degree of crystallinity and crystallites size of the samples were estimated by X-ray diffraction (XRD) (Thermo Fisher, Waltham, MA, USA) analysis using a PANalytical Empyrean diffractometer at room temperature, with a Cu X-ray tube (λ Cu Kα1 = 1.541874 Ǻ) operating in-line focusing, with programmable divergent slit on the incident side and a programmable anti-scatter slit mounted on the PIXcel3D detector on the diffracted side. The XRD diffraction patterns, recorded in the 2θ range of 20°–80°, were collected in a Bragg-Brentano geometry, with a scan step of 0.02° and a 255 s counting time per step. The lattice parameters were calculated using the HighScore Plus 3.0 e software and refined by the Rietveld method. The morphology of the samples was studied by scanning electron microscopy (SEM) using a Quanta Inspect F50 FEG (field emission gun) with 1.2 nm resolution (Thermo Fisher, Waltham, MA, USA). The bright field TEM images were obtained using a high resolution Titan Themis transmission electron microscope from Thermo Fisher Scientific (Waltham, MA, USA). The microscope operated in transmission mode at a voltage of 200 kV. UV-Vis diffused reflectance spectra were obtained using an Able Jasco V-560 spectrophotometer (PW de Meern, The Netherlands), with a scan speed of 200 nm/s, between 200 and 850 nm. The fluorescence spectra were measured using a Perkin Elmer LS 55 fluorescence spectrophotometer (Arkon, OH, USA). All the spectra were recorded with a scan speed of 200 nm/s between 350 and 800 nm, and with excitation and emission slit widths of 4, 7, and 10 nm, respectively. The used excitation wavelength was 320 nm.

### 2.4. Cellular Viability Assays

#### 2.4.1. MTT Assay

The biocompatibility of cerium-doped hydroxyapatite samples was evaluated by means of MTT (3-(4,5-dimethylthiazolyl)-2,5-diphenyltetrazolium bromide) assay (Vybrant^®^MTT Cell Proliferation Assay Kit, Thermo Fischer, Waltham, MA, USA), performed on human mesenchymal amniotic fluid stem cells (AFSC). The cells were grown in DMEM medium (Sigma-Aldrich, St. Louis, MO, USA) supplemented with 10% fetal bovine serum, 1% antibiotics (penicillin and streptomycin) (Sigma-Aldrich, St. Louis, MO, MI, USA), which was changed twice a week. The method is based on the growing of AFSC cells in 96-well plates, with a seeding density of 3000 cells/well in the presence of the obtained powders for 72 h. Afterwards, 15 mL (12 mm) of MTT was added to the cells, and next were incubated at 37 °C for 4 h. A solution of 1 mg sodium dodecyl sulphate in 10 mL HCl, 0.01 M was added to the first one and pipetted vigorously to solubilize the formed formazan crystals. After 1 h, the optical density (OD) of solubilized formazan is evaluated spectrophotometrically using a TECAN Infinite M200 spectrophotometer (Männedorf, Switzerland) at 570 nm.

#### 2.4.2. GSH-Glo Glutathione Assay

The oxidative stress assessment was evaluated using the GSH-Glo Glutathione Assay kit (Promega, WI, USA). For the GSH-Glo Assay analysis, AFSC were seeded at a density of 3000 cells in 300 µL of Dulbecco’s Modified Eagle’s medium (DMEM) supplemented with 10% fetal bovine serum and 1% antibiotics (penicillin, streptomycin/neomycin) in 96-well plates. After 24 h of seeding, cells are treated with the studied samples and then incubated for 72 h. To the obtained solution, 100 µL 1 × GSH-Glo Reagent was added, followed by incubation at 37 °C for 30 min and then 100 µL Luciferin Detection Reagent was added and incubated at 37 °C for an additional time of 15 min. The medium from the wells was well homogenized and then the plate was read on the luminometer (Microplate Luminometer Centro LB 960, Berthold, Germany) [37,38].

#### 2.4.3. Cell Morphology and Viability Evaluation—Fluorescence Microscopy

The biocompatibility of the obtained cerium-doped HAp powders with AFSC was evaluated using fluorescence microscopy with a RED CMTPX fluorophore (Thermo Fischer, Waltham, MA, USA), a cell tracker for long-term tracing of living cells. The CMTPX tracker was added in cell culture, priory treated with the synthesized nanoparticles. After 5 days, the viability and morphology of the AFSC was evaluated. The CMTPX fluorophore, at a concentration of 5 µM and incubated for 30 min, was added in the culture medium, to allow the dye penetration into the cells. Finally, the AFSC were washed with PBS and visualized by fluorescent microscopy. The photomicrographs were taken using an Olympus CKX 41 digital camera driven by CellSense Entry software (Olympus, Tokyo, Japan) [38,39].

## 3. Results and Discussion

### 3.1. FTIR Analysis

**Figure 1:**
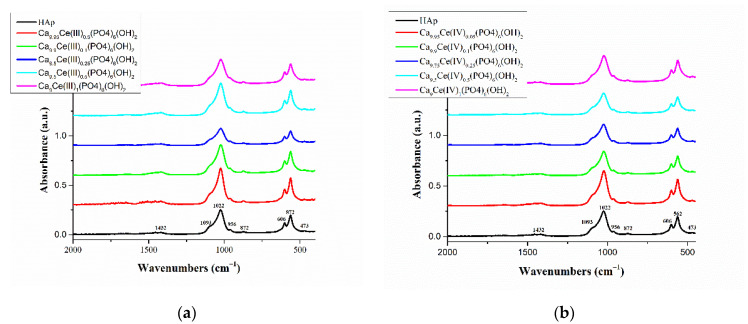
FTIR spectra of (**a**) hydroxyapatite (Hap) and Ca_10-x_Ce(III)_x_(PO_4_)_6_(OH)_2_; (**b**) hydroxyapatite (Hap) and Ca_10-x_Ce(IV)_x_(PO_4_)_6_(OH)_2_.

The FTIR spectroscopy was used to identify the characteristic functional groups present in pure Hap, Ce(III)-HAp and Ce(IV)-HAp. FTIR spectra of pure HAp, Ce(III)_x_HAp and Ce(IV)_x_HAp samples are shown in Figure 1a,b. The FTIR spectrum of pure HAp (black line from Figure 1a,b) shows all the bands reported in literature [40]. The bands in the region 3000–3400 cm^−1^ are due to the existence of bending and stretching modes of the OH-group. The bands around 1093, 1022, and 956 cm^−1^, respectively are due to the asymmetrical and symmetrical stretching modes of P–O bonds in the PO^3−^_4_ group [41]. The bands around 606, 562, and 473 cm^−1^, respectively are attributed to the bending mode of O–P–O phosphate bands [42]. The bands around 872 and 1432 cm^−1^ which appear in FTIR spectra of HAp indicate the presence of carbonate moieties in the prepared HAp powder [43].

Comparing the FTIR spectra from Figure 1a,b, it was found that the FTIR spectra of Ce(III)-doped HAp and Ce(IV)-doped HAp powders, respectively with various cerium concentrations are similar to that of pure HAp. In all FTIR spectra of Ca_10-x_Ce(III)x(PO_4_)_6_(OH)_2_ and Ca_10-x_Ce(IV)x(PO_4_)_6_(OH)_2_ samples, the absorption bands which appear around 872 and 1432 cm^−1^ are assigned to CO_3_^2−^. This can be attributed to the carbonate CO_3_^2−^ groups that replaced the PO_4_^3−^groups, indicating a reaction between HAp and carbon dioxide in the air [44]. The band intensity of PO_4_^3−^ bands at 1093–956 cm^−1^ were found to be decreased with the increase in the inclusion of Ce^3+^ or Ce^4+^ into the HAp lattice until the molar fraction of cerium (III) and cerium (IV) is 2.5%. Above this concentration the banding bands of PO_4_^3−^ increases. The replacement of calcium ions with cerium ions causes a change of bonding forces between the ions and leads to the weakening of the banding bands of O–P–O in the HAp structure [42]. The increasing concentration of Ce^3+^ and Ce^4+^ ions, which resulted in lowering the intensity of the bands, is associated with a decrease in HAp crystallinity. These results were further evidenced by the XRD analysis.

### 3.2. X-ray Analysis

**Figure 2:**
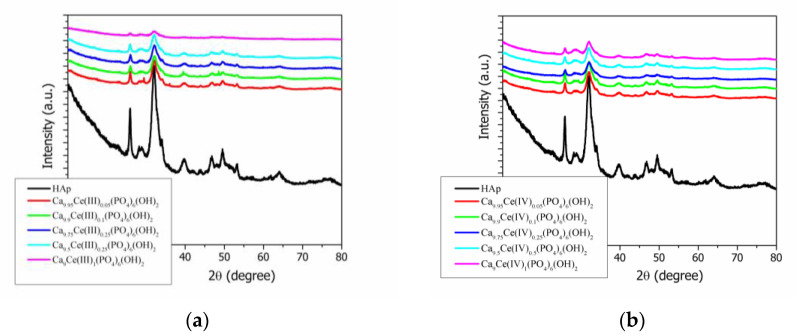
(**a**) X-ray diffraction patterns of hydroxyapatite (Hap) and Ca_10_−xCe(III)x(PO4)_6_(OH)_2_; (**b**) X-ray diffraction patterns of hydroxyapatite (Hap) and Ca_10_−xCe(IV)x(PO4)_6_(OH)_2_.

**Figure 3:**
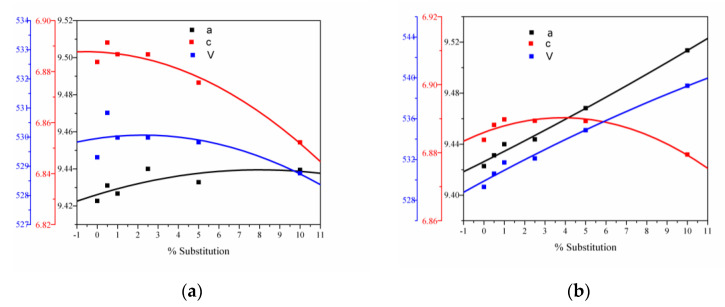
(**a**) Unit cell parameters versus substitution degree for Ca_10−x_Ce(III)_x_(PO_4_)_6_(OH)_2_; (**b**) unit cell parameters versus substitution degree for Ca_10−x_Ce(IV)_x_(PO_4_)_6_(OH)_2_.

The phase formation by the co-precipitation method of pure Hap, Ce(III)-doped hydroxyapatite, and Ce(IV)-doped Hap, respectively were shown in Figure 2a,b. The XRD spectra for pure and Ce-doped hydroxyapatite were compared and revealed only a pure and crystalline phase with well-defined diffraction peaks that match with the standard pattern of a hexagonal structure (according to ICDD PDF4+ card no. 00-068-0738) [45] with space group P6_3_/m, in agreement with literature data [46,47,48]. The XRD patterns from Figure 2a,b show a single phase calcium phosphate with all significant diffraction peaks: (002), (121), (112), (030), (022), (130), (222), (123), and (004). The degree of crystallinity and the crystallite size, calculated from the XRD results of pure Hap, Ce(III)-doped hydroxyapatite, and Ce(IV)-doped Hap, respectively were shown in Table 1 and Figure 3a,b. All the Ce(III)-doped hydroxyapatite and Ce(IV)-doped Hap patterns, respectively have shown decreases in the intensities of X-ray peaks with the increases in ion doping concentration level compared with pure Hap. 

There are some differences between Ce(III)-doped hydroxyapatite and Ce(IV)-doped Hap, respectively.

The average crystallite size of the pure Hap was 6.07 nm and for the cerium ions-doped Hap powders, a decrease in crystallite size with the increasing cerium content was observed. As can be seen from Table 2, the lattice parameters of the studied powders slowly decrease with the increasing content of the cerium cations. Some differences can be highlighted: (i) For all the samples doped with Ce^3+^ and Ce^4+^, a decrease in crystallite size with the increase in cerium content was observed. Only for a 2.5% Ce^3+^ substitution a small increase in crystallite size appears; (ii) the crystallinities were decreased with increases in cerium content due to the incorporation of cerium ions into the Hap lattice.

The values of unit cell parameters a, c, V, and the agreement indices of the Rietveld analysis (R_exp_, R_p_, R_wp_, and χ^2^) are shown in Table 2 and Figure 3a,b.

The lattice parameters a = b and c obtained from the XRD pattern of pure Hap shows a value of a = b = 9.42 Å and c = 6.88 Å, in agreement with literature data [49,50]. The changes in the lattice parameters are due to the ionic radius of ions: Ce^4+^ (0.97Å); Ce^3+^ (1.19 Å); compared with Ca^2+^ is 1Å. When Ca^2+^ ions are replaced by larger ions, such as Ce^3+^, the most significant change in lattice parameters is an increase in the a and c lattice parameters [47]. The differences observed for smaller ions than Ca^2+^ ion, such as Ce^4+^ are caused by their small differences in the ionic radius. When an ionic radius is greater than others, a slow lattice expansion is observed. For the same rare earth ion, the differences at a low concentration are not observed, but a higher substitution degree induces significant changes in the unit cell parameters. The metallic ion exchanges can lead to minimal distortion of bonds in the Hap structure [51,52]. The lattice of the Hap contains two Ca atoms with different crystal configurations: Ca(1)—nine coordinated with an ionic radius of 0.118 and Ca(2)—octahedral coordinated with an ionic radius of 0.106 nm, respectively [53]. From the literature, it is known that ions with larger ionic radius and smaller charge preferentially occupy the Ca(1) site, while ions with a smaller ionic radius and greater charge occupy the Ca(2) site [54,55]. In the present research, the differences in the ionic radius of the substitution ions suggest that the Ce^3+^ ion favors the substitution in the Ca(1) site, and the Ca(2) site would be occupied by Ce^4+^ ions, since it has a smaller ionic radius than Ce^3+^.

**Figure 4:**
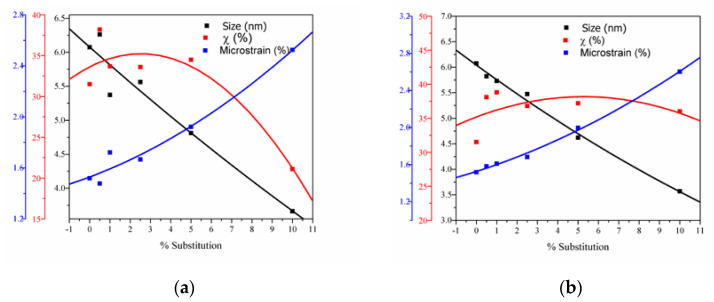
(**a**) The estimated crystallite size, lattice microstrain, and degree of crystallinity for Ca_10−x_Ce(III)_x_(PO_4_)_6_(OH)_2_; (**b**) the estimated crystallite size, lattice microstrain, and degree of crystallinity for Ca_10−x_Ce(IV)_x_(PO_4_)_6_(OH)_2_.

The variation of lattice microstrain values, crystallite size and crystallinity degree with cerium ions content are shown in Figure 4.

The crystallinity degree decreases with the increase in cerium concentration due to the slower kinetics of solid solution formation with multiple cations on the Ca^2+^ site [56].

### 3.3. SEM Analysis

**Figure 5:**
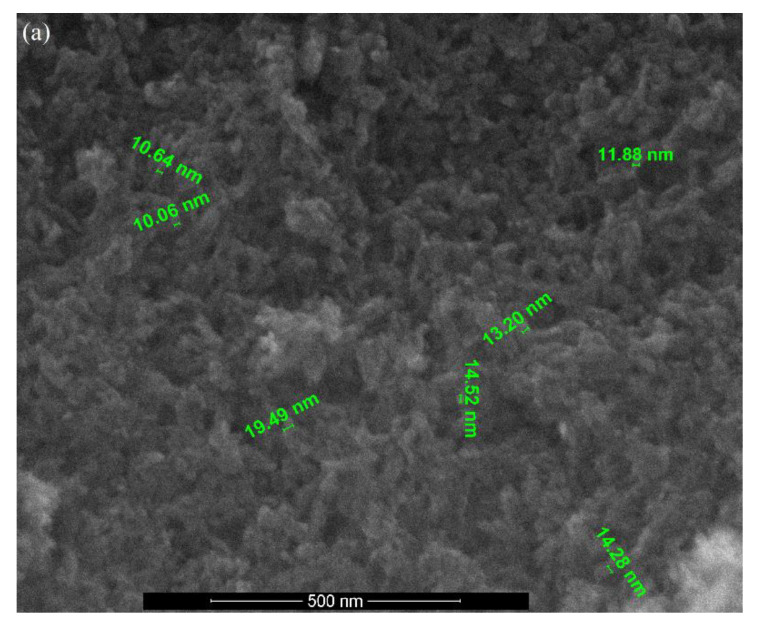

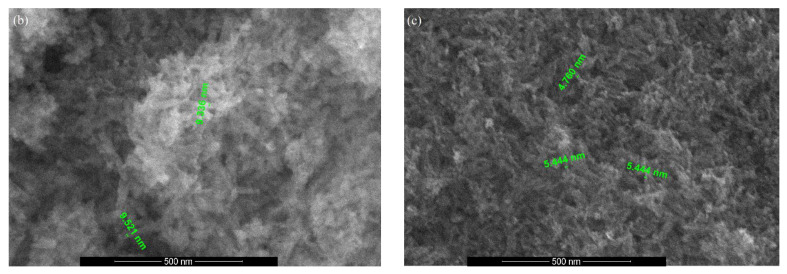
The SEM image of (**a**) pure Hap; (**b**) Ca_9.5_Ce(III)_0.5_(PO_4_)_6_(OH)_2_ and (**c**) Ca_9.5_Ce(IV)_0.5_(PO_4_)_6_(OH)_2_.

The surface morphologies of pure Hap, Ce(III)-doped hydroxyapatite and Ce(IV)-doped hydroxyapatite with various concentrations were shown in Figure 5. The SEM image of pure Hap (Figure 5a) reveals that the particles are in the spherical shape with a dense nature, and the size was found to be in the range of 10–20 nm.

By doping with Ce(III) and Ce(IV) ions, respectively a little influence can be observed in the morphology of substituted Hap. Figure 5b,c show the SEM images of Ca_9.5_Ce(III)_0.5_(PO_4_)_6_(OH)_2_ and Ca_9.5_Ce(IV)_0.5_(PO_4_)_6_(OH)_2_. Both samples exhibit the morphologies of particles of spherical shapes with a tendency of forming agglomeration. The observed particle sizes range from about 9 to 10 nm for Ce(III)-doped Hap, and 4 to 5 nm for Ce(IV)-doped Hap, respectively.

### 3.4. TEM Analysis

**Figure 6:**
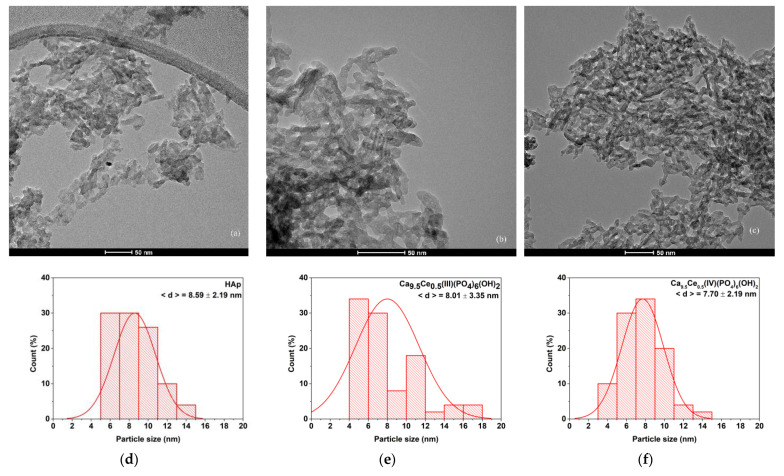
The TEM image of (**a**) pure HAp; (**b**) Ca_9.5_Ce(III)_0.5_(PO_4_)_6_(OH)_2_ and (**c**) Ca_9.5_Ce(IV)_0.5_(PO_4_)_6_(OH)_2_. Particle size distribution of obtained materials for (**d**) pure HAp; (**e**) Ca_9.5_Ce(III)_0.5_(PO_4_)_6_(OH)_2_ and (**f**) Ca_9.5_Ce(IV)_0.5_(PO_4_)_6_(OH)_2_.

In order to further evaluate nanodimensions of Hap, TEM investigations were made. Thus, Figure 6 shows the bright field TEM of pure Hap, Ca_9.5_Ce(III)_0.5_(PO_4_)_6_(OH)_2_ and Ca_9.5_Ce(IV)_0.5_(PO_4_)_6_(OH)_2_, and particle size distributions.

From the bright field TEM images obtained on all three samples, we can observe the rod-like morphology of the nanoparticles, typical for HAP. The size distributions obtained on the width of the particles reveal the monomodal distribution of HAp. Ce doping has the effect of reducing the particle size in both cases. The particle size distribution varies from 8.59 to 7.70 nm. The length of the Hap particles are in the range of 10 to 25 nm.

### 3.5. UV-Vis and PL Spectra

**Figure 7:**
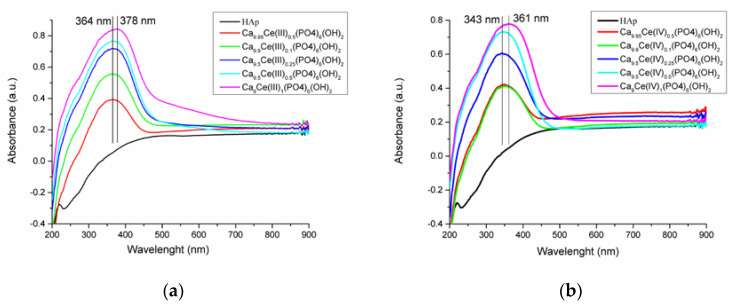
UV-Vis absorption spectra of (**a**) Ca_10_−_x_Ce(III)_x_(PO_4_)_6_(OH)_2_ and (**b**) Ca_10_−_x_Ce(IV)_x_(PO_4_)_6_(OH)_2_ with different concentrations.

The UV-Vis spectra of the two cerium(III)-doped hydroxyapatite and cerium(IV)-doped hydroxyapatite, respectively are shown in Figure 7a,b. A general trend was observed for all the studied compounds: An increase in the intensity of absorption peaks and a broadening of them with increases in cerium ions concentrations in HAp. 

The UV-Vis absorption spectra of Ca_10-x_Ce(III)x(PO_4_)_6_(OH)_2_ and Ca_10-x_Ce(IV)x(PO_4_)_6_(OH)_2_, with different concentrations represented in Figure 7a,b are similar.

The UV-Vis spectra of Ca_10-x_Ce(III)x(PO_4_)_6_(OH)_2_ has maxima which covers a wavelength range from 364 to 378 nm, and for Ca_10-x_Ce(IV)x(PO_4_)_6_(OH)_2_ from 343 to 361 nm, respectively. The intensities of this maxima increase with the increasing cerium content as Ce ions display strong UV absorbtion [57]. Pure hydroxyapatite presents no absorbtion band, therefore the UV absorbtion band of the doped powders can be attributed to the presence of Ce ions.

**Figure 8:**
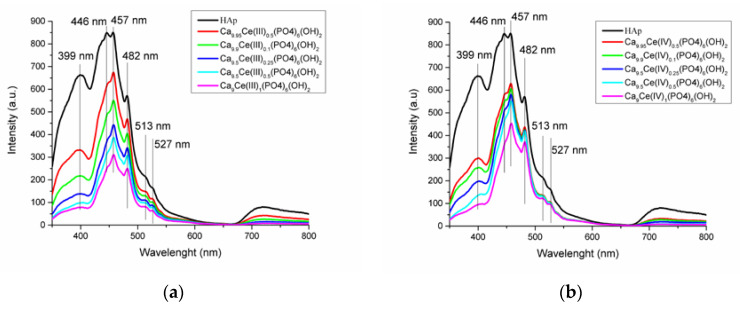
Room-temperature photoluminescence spectra of (**a**) Ca_10−x_Ce(III)_x_(PO_4_)_6_(OH)_2_ and (**b**) Ca_10−x_Ce(IV)_x_(PO_4_)_6_(OH)_2_ with different concentrations.

Figure 8a,b presents the photoluminescence emission spectra of Ca_10-x_Ce(III)_x_(PO_4_)_6_(OH)_2_ and Ca_10-x_Ce(IV)_x_(PO_4_)_6_(OH)_2_ samples excited with 320 nm wavelength. It was observed that the intensities of peaks decrease with the increasing cerium ions content. All emission peaks which appear at 399, 446, 457, 482, 513, and 527 nm are associated with various oxygen vacancies, interstitial or oxygen antisites. 

### 3.6. MTT Assay

**Figure 9:**
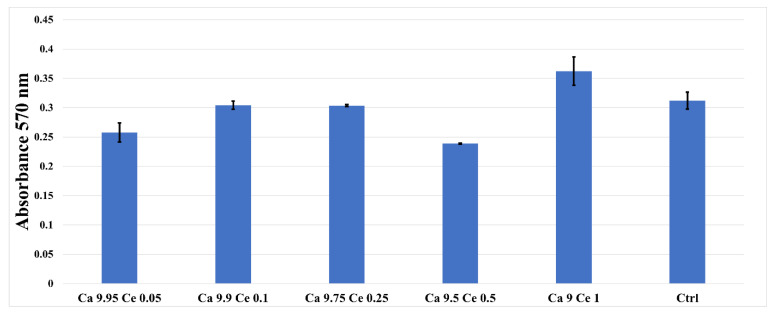
MTT assay showing the viability of AFSC in the presence of the cerium-doped HAp nanomaterials and control (cell only).

The MTT assay revealed that the synthesized Ce (III)-doped HAp and Ce(IV)-doped HAp nanoparticles reveal no cytotoxic effect, the absorption values being close, lower or higher compared to the control sample or any toxic effects even at higher concentrations (Figure 9). The tested samples stimulated cellular metabolism, with a significant increase in proliferation compared to the control at 72 h.

**Figure 10:**
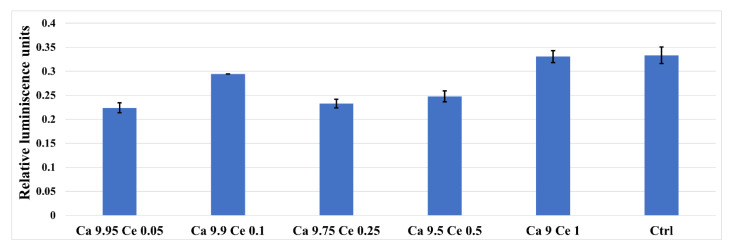
GSH assay showing the oxidative stress of AFSC in the presence of the cerium-doped HAp nanomaterials and control (cell only).

In the presence of cerium-doped nanomaterials, the glutathione a marker of oxidative stress, is able to prevent cellular damage caused by reactive oxygen species, such as free radicals, peroxides, lipid peroxides, and heavy metals (Figure 10), as well as responds similarly to the control cells, indicating that the analyzed materials do not induce cellular stress.

**Figure 11:**
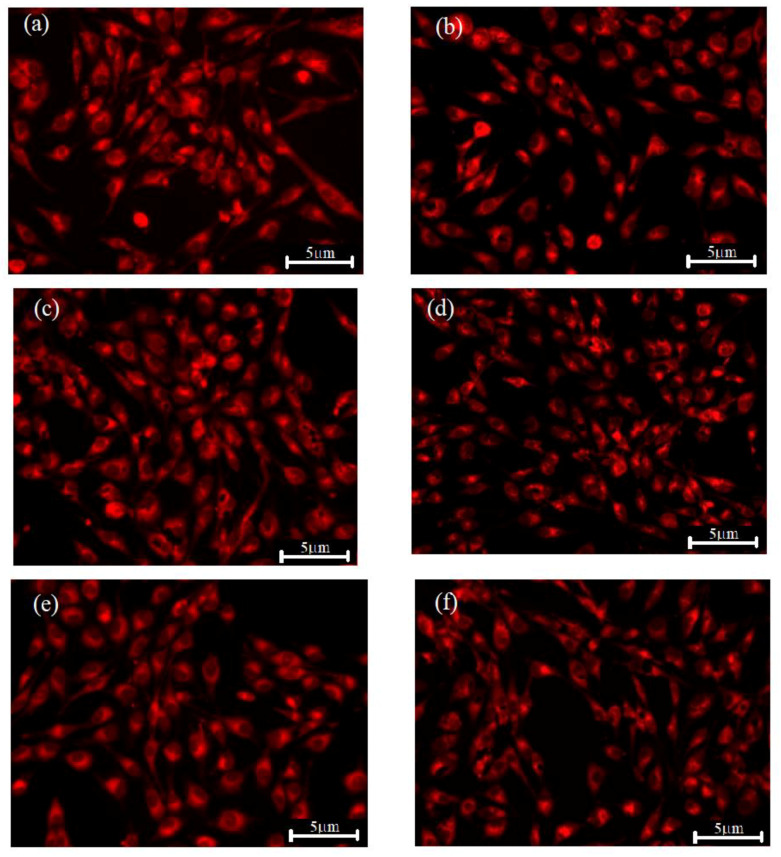
Fluorescence images of Ca_10-x_Ce_x_(PO_4_)_6_(OH)_2_ samples colored with CMTPX fluorophore (**a**) control sample, (**b**) x = 0.5; (**c**) x = 1; (**d**) x = 2.5; (**e**) x = 5; (**f**) x = 10%.

Fluorescence microscopy (Figure 11) shows that AFSC cells are viable and cerium-doped HAp nanomaterial samples have no cytotoxic effect, thus confirming the biochemical results. Therefore, the fluorescent synthesized nanoparticles could be used as fluorescent probing samples for cellular imaging and also as an antioxidant agent against oxidative stress-induced cell damages.

**Figure 12:**
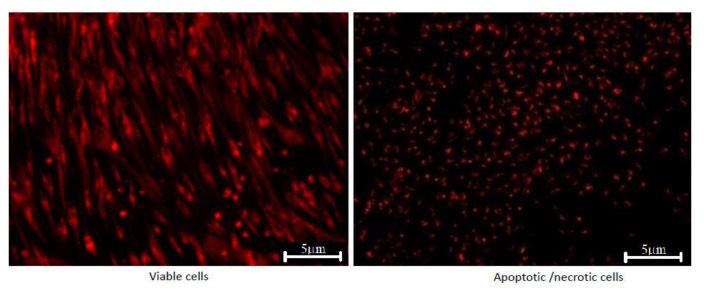
Fluorescent microscopy images of cerium-doped HAp powders (viable cells) and negative control sample.

After 5 days in the presence of cerium-doped HAp powders, the AFSC showed normal morphology with a fibroblast-like characteristic appearance. Fluorescence images showed that the AFSC were viable, no dead cells or cell fragments were observed. Moreover, the cells formed filopodia to move and establish contacts with neighboring cells, suggesting that AFSC exhibited an active phenotype. Furthermore, the picture of a negative control (dead cells) for the fluorescent microscopy images was represented in Figure 12. The negative control is represented by the AFSC treated with H_2_O_2_, 10 mm.

## 4. Conclusions

Pure hydroxyapatite, Ce^3+^-doped HAp, and Ce^4+^-doped HAp were prepared using the co-precipitation method. Analysis of pure HAp and cerium-doped HAp using spectral and scanning electron microscopy were performed. The FTIR spectroscopy and XRD diffraction analysis confirmed the formation of HAp. The results of FTIR spectroscopy and XRD analysis demonstrate that the calcium ions were successfully replaced by cerium ions in the hydroxyapatite crystal lattice. The SEM micrographs showed typical spherical morphologies for all the studied samples. The Ce^3+^-doped HAp and Ce^4+^-doped HAp samples exhibit a high fluorescent emission peak. The MTT assay showed that with cerium doping no cytotoxic effect was observed when the dose cells were incubated with fluorescent nanoparticles. Therefore, the obtained samples in this present research could be used as a fluorescent probing for cellular imaging.

## Figures and Tables

**Table 1:** Calculated crystallite size (D), internal strains (S) values, and degree of crystallinity (χ_c_) of pure Hap, cerium (III)-doped hydroxyapatite and cerium (IV)-doped hydroxyapatite with various concentrations.

Sample	D/nm	S/%	χ_c_/%
Hap	6.1 ± 0.8	1.5 ± 0.5	31.5
Ca_10−x_Ce(III)_x_(PO_4_)_6_(OH)_2_			
Ca_9.95_Ce(III)_0.05_(PO_4_)_6_(OH)_2_	6.3 ± 0.7	1.5 ± 0.6	38.2
Ca_9.9_Ce(III)_0.1_(PO_4_)_6_(OH)_2_	5.4 ± 0.6	1.7 ± 0.6	33.7
Ca_9.75_Ce(III)_0.25_(PO_4_)_6_(OH)_2_	5.6 ± 0.5	1.7 ± 0.6	33.6
Ca_9.5_Ce(III)_0.5_(PO_4_)_6_(OH)_2_	4.8 ± 0.6	1.9 ± 0.7	34.5
Ca_9_Ce(III)_1_(PO_4_)_6_(OH)_2_	3.7 ± 0.6	2.5 ± 0.9	21.1
Ca_10−x_Ce(IV)_x_(PO_4_)_6_(OH)_2_			
Ca_9.95_Ce(IV)_0.05_(PO_4_)_6_(OH)_2_	5.8 ± 0.8	1.6 ± 0.5	38.0
Ca_9.9_Ce(IV)_0.1_(PO_4_)_6_(OH)_2_	5.7 ± 0.8	1.6 ± 0.6	38.8
Ca_9.75_Ce(IV)_0.25_(PO_4_)_6_(OH)_2_	5.5 ± 0.8	1.7 ± 0.5	36.8
Ca_9.5_Ce(IV)_0.5_(PO_4_)_6_(OH)_2_	4.6 ± 0.8	2.0 ± 0.6	37.2
Ca_9_Ce(IV)_1_(PO_4_)_6_(OH)_2_	3.6 ± 0.5	2.6 ± 0.9	36.0

**Table 2:** Unit cell parameters a, c, V, and the agreement indices for hydroxyapatite cerium (III)-doped hydroxyapatite and cerium (IV)-doped hydroxyapatite with various concentrations.

Sample	a [Å]	c [Å]	V [Å^3^]	R_exp_	R_p_	R_wp_	χ^2^
Hap	9.423 ± 0.003	6.884 ± 0.003	529.309	3.105	4.506	5.704	3.375
Ca_10−x_Ce(III)_x_(PO_4_)_6_(OH)_2_							
Ca_9.95_Ce(III)_0.05_(PO_4_)_6_(OH)_2_	9.431 ± 0.003	6.891 ± 0.002	530.832	3.660	4.186	5.724	2.447
Ca_9.9_Ce(III)_0.1_(PO_4_)_6_(OH)_2_	9.427 ± 0.004	6.887 ± 0.003	529.987	3.736	3.985	5.182	1.924
Ca_9.75_Ce(III)_0.25_(PO_4_)_6_(OH)_2_	9.440 ± 0.003	6.887 ± 0.003	531.485	3.680	3.934	5.099	1.920
Ca_9.5_Ce(III)_0.5_(PO_4_)_6_(OH)_2_	9.433 ± 0.005	6.876 ± 0.004	529.823	3.601	4.544	6.005	2.781
Ca_9_Ce(III)_1_(PO_4_)_6_(OH)_2_	9.439 ± 0.010	6.852 ± 0.008	528.761	5.011	4.929	6.126	1.494
Ca_10−x_Ce(IV)_x_(PO_4_)_6_(OH)_2_							
Ca_9.95_Ce(IV)_0.05_(PO_4_)_6_(OH)_2_	9.431 ± 0.0030	6.888 ± 0.0023	530.608	3.687	4.143	5.452	2.187
Ca_9.9_Ce(IV)_0.1_(PO_4_)_6_(OH)_2_	9.440 ± 0.003	6.890 ± 0.003	531.711	3.662	4.351	5.790	2.501
Ca_9.75_Ce(IV)_0.25_(PO_4_)_6_(OH)_2_	9.444 ± 0.004	6.889 ± 0.003	532.104	3.599	4.682	6.158	2.928
Ca_9.5_Ce(IV)_0.5_(PO_4_)_6_(OH)_2_	9.468 ± 0.006	6.889 ± 0.005	534.867	3.773	5.942	7.942	4.431
Ca_9_Ce(IV)_1_(PO_4_)_6_(OH)_2_	9.514 ± 0.0108	6.879 ± 0.009	539.224	3.772	7.137	9.886	6.868

## Data Availability

Not applicable.

## References

[1] Dorozhkin S.V. (2011). Calcium orthophosphates: Occurrence, properties, biomineralization, pathological calcification and biomimetic applications. Biomatter.

[2] Oryan A., Alidadi S., Moshiri A., Maffulli N. (2014). Bone regenerative medicine: Classic options, novel strategies, and future directions. Journal of orthopaedic surgery and research. J. Orthop. Surg. Res..

[3] Chung R.J. (2011). Study of hydroxyapatite nano composites with photoluminescence properties. Biomed. Eng. Appl. Basis Commun..

[4] Dasgupta S., Banerjee S.S., Bandyopadhyay A., Bose S. (2010). Zn- and Mg-doped hydroxyapatite nanoparticles for controlled release of protein. Langmuir.

[5] Talal A., Hamid S.K., Khan M., Khan A.S. (2019). Structure of Biological Apatite: Bone and Tooth. Bone and Tooth.

[6] Dorozhkin S.V. (2013). Nanodimensional and nanocrystalline hydroxyapatite and other calcium orthophosphates. Hydroxyapatite Synth. Prop. Appl..

[7] Ma M.Y., Zhu Y.J., Li L., Cao S.W. (2008). Nanostructured porous hollow ellipsoidal capsules of hydroxyapatite and calcium silicate: Preparation and application in drug delivery. J. Mater. Chem..

[8] Li L., Liu Y., Tao J., Zhang M., Pan H., Xu X., Tang R. (2008). Surface modification of hydroxyapatite nanocrystallite by a small amount of terbium provides a biocompatible fluorescent probe. J. Phys. Chem. C.

[9] Chane-Ching J.Y., Lebugle A., Rousselot I., Pourpoint A., Pellé F. (2007). Colloidal synthesis, and characterization of monocrystalline apatite nanophosphors. J. Mater. Chem..

[10] Yang Z., Huang Y., Chen S.T., Zhao Y.Q., Li H.L., Hu Z.A. (2005). Template synthesis of highly ordered hydroxyapatite nanowire arrays. J. Mater. Sci..

[11] Liu C., Ji X., Cheng G. (2007). Template synthesis and characterization of highly ordered lamellar hydroxyapatite. Appl. Surf. Sci..

[12] Liu Y., Hou D., Wang G. (2004). A simple wet chemical synthesis and characterization of hydroxyapatite nanorods. Mater. Chem. Phys..

[13] Ripamonti U. (1991). The morphogenesis of bone in replicas of porous hydroxyapatite obtained from conversion of calcium carbonate exoskeletons of coral. J. Bone Jt. Surg. Ser. A.

[14] Ripamonti U., Ma S.-S., van der Heever B., Reddi A.H. (1992). Osteogenin, a bone morphogenetic protein, absorbed on porous hydroxyapatite substrata, induces rapid bone differentiation in calvarial defects of adults primates. Plast. Reconstr. Surg..

[15] Barbeck M., Udeabor S., Lorenz J., Schlee M., Holthaus M.G., Raetscho N., Choukroun J., Sader R., Kirkpatrick C.J., Ghanaati S. (2014). High-Temperature sintering of xenogeneic bone substitutes leads to increased multinucleated giant cell formation: In vivo and preliminary clinical results. J. Oral Implantol..

[16] Wei J.Q., Liu Y., Zhang X.H., Liang W.W., Zhou T.F., Zhang H., Deng X.L. (2017). Enhanced critical-sized bone defect repair efficiency by combining deproteinized antler cancellous bone and autologous BMSCs. Chin. Chem. Lett..

[17] Pramanik S., Agarwal A.K., Rai K.N., Garg A. (2007). Development of high strength hydroxyapatite by solid-state-sintering process. Ceram. Int..

[18] Sadat-Shojai M., Khorasani M.T., Dinpanah-Khoshdargi E., Jamshidi A. (2013). Synthesis methods for nanosized hydroxyapatite with diverse structures. Acta Biomater..

[19] Pham V.H., Van H.N., Tam P.D., Ha H.N.T. (2016). A novel 1540 nm light emission from erbium doped hydroxyapatite/β-tricalcium phosphate through co-precipitation method. Mater. Lett..

[20] Mobasherpour I., Heshajin M.S., Kazemzadeh A., Zakeri M. (2007). Synthesis of nanocrystalline hydroxyapatite by using precipitation method. J. Alloys Compd..

[21] Bose S., Saha S.K. (2003). Synthesis of hydroxyapatite nanopowders via sucrose-templated Sol-Gel method. J. Am. Ceram. Soc..

[22] Bigi A., Boanini E., Rubini K. (2004). Hydroxyapatite gels and nanocrystals prepared through a sol-gel process. J. Solid State Chem..

[23] Asri R.I.M., Harun W.S.W., Hassan M.A., Ghani S.A.C., Buyong Z. (2016). A review of hydroxyapatite-based coating techniques: Sol-gel and electrochemical depositions on biocompatible metals. J. Mech. Behav. Biomed. Mater..

[24] Türk S., Altınsoy I., ÇelebiEfe G., Ipek M., Özacar M., Bindal C. (2017). Microwave–Assisted biomimetic synthesis of hydroxyapatite using different sources of calcium. Mater. Sci. Eng. C.

[25] Hu C., Aindow M., Wei M. (2017). Focused ion beam-sectioning studies of biomimetic hydroxyapatite coatings on Ti-6Al-4V substrates. Surf. Coat. Technol..

[26] Yokota T., Ito R., Shimizu Y., Honda M., Aizawa M. (2017). Fabrication of sodium-substituted hydroxyapatite ceramics via ultrasonic spray-pyrolysis route and their material properties. KEM Key Eng. Mater..

[27] Cho J.S., Lee J.C., Rhee S.H. (2016). Effect of precursor concentration and spray pyrolysis temperature upon hydroxyapatite particle size and density. J. Biomed. Mater. Res. Part B Appl. Biomater..

[28] Consani S., Balić-Žunić T., Cardinale A.M., Sgroi W., Giuli G., Carbone C. (2018). A novel synthesis routine for woodwardite and its affinity towards light (La, Ce, Nd) and heavy (Gd and Y) rare earth elements. Materials.

[29] Gad S.C. (2014). Cerium. Encyclopedia of Toxicology.

[30] Kolmas J., Groszyk E., Kwiatkowska-Rózycka D. (2014). Substituted hydroxyapatite with antibacterial properties: A review article. BioMed Res. Int..

[31] Chen M.H., Yoshioka T., Toshiyuki I., Hanagata N., Lin F.H., Tanaka J. (2014). Photoluminescence and doping mechanism of theranostic Eu3+/Fe3+ dual-doped hydroxyapatite nanoparticles. Sci. Technol. Adv. Mater..

[32] Yingguang L., Zhuoru Y., Jiang C. (2007). Preparation, characterization and antibacterial property of cerium substituted hydroxyapatite nanoparticles. J. Rare Earths.

[33] Zhou G., Li Y., Zheng B., Wang W., Gao J., Wei H., Li S., Wang S., Zhang J. (2014). Cerium oxide nanoparticles protect primary osteoblasts against hydrogen peroxide induced oxidative damage. Micro. Nano Lett..

[34] Leonelli C., Lusvardi G., Malavasi G., Menabue L., Tonelli M. (2003). Synthesis and characterization of cerium-doped glasses and in vitro evaluation of bioactivity. J. Non Cryst. Solids.

[35] Kaygili O., Dorozhkin S.V., Keser S. (2014). Synthesis and characterization of Ce-substituted hydroxyapatite by sol-gel method. Mater. Sci. Eng. C.

[36] Peng B., Song K., Wang H., Zhang S., Su W., Cheng Z. (2018). Investigation on Ce^3+^ luminescence from different crystallographic sites, self energy transfer and abnormal thermal stability of nitrided Ba_9_Y_2_Si_6_O_24_: Ce^3+^ phosphor for W-LEDs. Ceram. Int..

[37] Andronescu E., Predoi D., Neacsu I.A., Paduraru A.V., Musuc A.M., Trusca R., Oprea O., Tanasa E., Vasile O.R., Nicoara A.I. (2019). Photoluminescent Hydroxylapatite: Eu3+ Doping Effect on Biological Behaviour. Nanomaterials.

[38] Nicoara A.I., Ene V.L., Voicu B.B., Bucur M.A., Neacsu I.A., Vasile B.S., Iordache F. (2020). Biocompatible Ag/Fe-Enhanced TiO_2_ Nanoparticles as an Effective Compound in Sunscreens. Nanomaterials.

[39] Paduraru A., Ghitulica C., Trusca R., Surdu V.A., Neacsu I.A., Holban A., Bîrca A., Iordache F., Vasile B.S. (2019). Antimicrobial Wound Dressings as Potential Materials for Skin Tissue Regeneration. Materials.

[40] Dhand V., Rhee K.Y., Park S.J. (2014). The facile and low temperature synthesis of nanophase hydroxyapatite crystals using wet chemistry. Mater. Sci. Eng. C.

[41] Yuan Q., Qin C., Wu J., Xu A., Zhang Z., Liao J., Lin S., Ren X., Zhang P. (2016). Synthesis and characterization of Cerium-doped hydroxyapatite/polylactic acid composite coatings on metal substrates. Mater. Chem. Phys..

[42] Feng Z., Liao Y., Ye M. (2005). Synthesis and structure of cerium-substituted hydroxyapatite. J. Mater. Sci. Mater. Med..

[43] Priyadarshini B., Anjaneyulu U., Vijayalakshmi U. (2017). Preparation and characterization of sol-gel derived Ce^4+^ doped hydroxyapatite and its in vitro biological evaluations for orthopedic applications. Mater. Des..

[44] Murugan R., Ramakrishna S. (2006). Production of ultra-fine bioresorbable carbonated hydroxyapatite. Acta Biomater..

[45] Veselinovic L., Karanovic L., Stojanovic Z., Bracko I., Markovic S., Ignjatovic N., Uskokovic D. (2010). Crystal structure of cobalt-substituted calcium hydroxyapatite nanopowders prepared by hydrothermal processing. J. Appl. Crystallogr..

[46] Lima T.A.R.M., Valerio M.E.G. (2018). X-ray absorption fine structure spectroscopy and photoluminescence study of multifunctional europium (III)-doped hydroxyapatite in the presence of cationic surfactant medium. J. Lumin..

[47] Elliott J.C. (1994). Structure and Chemistry of the Apatites and Other Calcium Orthophosphates.

[48] Suzuki T., Hatsushika T., Miyake M. (1982). Synthetic hydroxyapatites as inorganic cation exchangers. Part2. J. Chem. Soc. Faraday Trans..

[49] Gaines R.V., Skinner H.C., Foord E.E., Mason B., Rosenzweig A. (1997). Dana’s New Mineralogy.

[50] Klein C., Dutrow B. (2008). Mineral Science.

[51] Shannon R.D. (1976). Revised effective ionic radii and systematic studies of inter atomic distances in halides and chalcogenides. Acta Crystallogr. Sect. A Found. Crystallogr..

[52] Rameshbabu N., Sampath Kumar T.S., Prabhakar T.G., Sastry V.S., Murty K.V.G.K., Prasad Rao K. (2007). Antibacterial nanosized silver substituted hydroxyapatite: Synthesis and characterization. J. Biomed. Mater. Res. A.

[53] Phataia P., Futalan C.M., Utara S., Khemthong P., Kamonwannasit S. (2018). Structural characterization of cerium-doped hydroxyapatite nanoparticles synthesized by an ultrasonic-assisted sol-gel technique. Results Phys..

[54] Ellis D.E., Terra J., Warschkow O., Jiang M., González G.B., Okasinski J.S., Bedzyk M.J., Rossi A.M., Eon J.G. (2006). A theoretical and experimental study of lead substitution in calcium hydroxyapatite. Phys. Chem. Chem. Phys..

[55] Getman E.I., Ignatov A.V., Loboda S.N., Abdul Jabar M.A.B., Zhegailo A.O., Gluhova A.S. (2011). Substitution of samarium for strontium on the structure of hydroxyapatite. Funct. Mater..

[56] Cacciotti I., Antoniac I. (2016). Cationic and Anionic Substitutions in Hydroxyapatite. Handbook of Bioceramics and Biocomposites.

[57] Huang W.J., Mao Z., Chen L., Chi Y.L., Jiang H., Zimba B.L., Xiong G.X., Wu Q.Z. (2018). Synthesis and characterisation of fluorescent and biocompatible hydroxyapatite nanoparticles with cerium doping. Micro Nano Lett..

